# Biodegradation of high concentrations of halomethanes by a fermentative enrichment culture

**DOI:** 10.1186/s13568-014-0048-5

**Published:** 2014-06-14

**Authors:** Huifeng Shan, Han Wang, Rong Yu, Priya Jacob, David L Freedman

**Affiliations:** 1 PeroxyChem Environmental Solutions (East Asia), Room 5B16, West Wing, Hanwei Plaza, 7 Guanghua Road, Chaoyang District, Beijing 100004, China; 2 Department of Environmental Engineering and Earth Sciences, Clemson University, Clemson, 29634-0919, SC, USA

**Keywords:** Chloroform, Carbon tetrachloride, Trichlorofluoromethane, Vitamin B12, Bioremediation

## Abstract

A fermentative enrichment culture (designated DHM-1) that grows on corn syrup was evaluated for its ability to cometabolically biodegrade high concentrations of chloroform (CF), carbon tetrachloride (CT), and trichlorofluoromethane (CFC-11). When provided with corn syrup and vitamin B_12_ (0.03 mol B_12_ per mol CF), DHM-1 grew and biodegraded up to 2,000 mg/L of CF in 180 days, with only minor transient accumulation of dichloromethane and chloromethane. CT (15 mg/L) and CFC-11 (25 mg/L) were also biodegraded without significant accumulation of halomethane daughter products. The rate of CF biodegradation followed a Michaelis-Menten-like pattern with respect to the B_12_ concentration; one-half the maximum rate (66 mg CF/L/d) occurred at 0.005 mol B_12_ per mol CF. DHM-1 was able to biodegrade 500 mg/L of CF at an inoculum level as low as 10^−8^ mg protein/L. The highest rate of CF biodegradation occurred at pH 7.7; activity decreased substantially below pH 6.0. DHM-1 biodegraded mixtures of CT, CFC-11, and CF, although CFC-11 inhibited CF biodegradation. Evidence for compete defluorination of CFC-11 was obtained based on a fluoride mass balance. Overall, the results suggest that DHM-1 may be effective for bioaugmentation in source zones contaminated with thousands of milligrams per liter of CF and tens of milligrams per liter of CT and CFC-11.

## Introduction

Over the past two decades, substantial progress has been made in use of bioremediation for treating halogenated solvents in groundwater. Nevertheless, in situ bioremediation strategies for groundwater with high concentrations of halogenated methanes such as carbon tetrachloride (CT), chloroform (CF), and trichlorofluoromethane (CFC-11) are still lacking. Among these compounds, CF is often the focal point for evaluating the feasibility of bioremediation because of its high toxicity to most microbes. For instance, inhibition of chlororespiration of chloroethenes by CF is a concern for sites co-contaminated with CF, and can only be overcome by removing the CF first (Bagley et al. [2000]). CF and CT rank highly on the Agency for Toxic Substances and Disease Registry based on their frequency, toxicity, and potential for human exposure at National Priority List sites (ATSDR [2013]). Although not ranked by ATSDR, CFC-11 is often a co-contaminant with CT and CF. For example, approximately 500 mg/L CF, 26 mg/L CFC-11 and 10 mg/L CT were detected in the source zone at a former industrial site (Shan et al. [2010b]). Bioremediation of mixtures of high concentrations of halomethanes is especially challenging.

Most previous research on bioremediation of CT, CF and CFC-11 at elevated concentrations focused on individual compounds. For example, in the presence of vitamin B_12,_ a fermentative culture grown on dichloromethane (DCM) transformed up to 270 mg/L CF (Becker and Freedman [1994]) and a sulfate reducing enrichment culture developed from anaerobic digester sludge transformed up to 350 mg/L CT (Freedman et al. [1995]). The highest concentration of CFC-11 evaluated previously was 2.2 mg/L and resulted in accumulation of dichlorofluoromethane (HCFC-21), which is not an acceptable endpoint (Krone and Thauer [1992]). Although bioaugmentation continues to mature as an option for treatment of chlorinated ethenes (ESTCP 2005), there has been less progress with halogenated methanes. SDC-9™ biodegrades CT and CF, but it has been evaluated at relatively low concentrations, i.e., 2.3 mg/L CT and 3.2 mg/L CF (ESTCP 2005). Recently, *Dehalobacter* spp. have been described that reductively dechlorinate up to approximately 60 mg/L of CF to DCM via organohalide respiration (Grostern et al. [2010]; Lee et al. [2012]). DCM is not an acceptable endpoint, but microbes that use DCM as a sole carbon and energy source have been reported (Freedman and Gossett [1991]; Justicia-Leon et al. [2012]; Mägli et al. [1998]); Lee et al. ([2012]) described a mixture of *Dehalobacter* spp. that reduced 50 mg/L of CF to DCM, which was subsequently fermented to acetate, CO_2_, and H_2_; evaluation of higher CF concentrations was not reported.

In a microcosm study of the abovementioned industrial site, we demonstrated that bioaugmentation is a potentially feasible remediation option for the highest concentration areas of the plume, containing CT, CFC-11, and CF (Shan et al. [2010b]). Cometabolic biodegradation of the halomethanes was accomplished via addition of a fermentative enrichment culture that grows on corn syrup, along with vitamin B_12_ at a dose of 0.03 mol B_12_ per mol of CT + CFC-11 + CF. Addition of only corn syrup + B_12_ was also effective, but took twice as long. Accumulation of DCM, chloromethane (CM), HCFC-21, and chlorofluoromethane (HCFC-31) was minor. Following numerous transfers of the culture (designated DHM-1) in mineral salts medium (MSM) amended with corn syrup, CF, and B_12_, we demonstrated that DHM-1 grows equally well in the presence or absence of 500 mg/L of CF (Shan et al. [2010a]). This was promising from the perspective that bioaugmentation cultures should be able to grow in the presence of the contaminants.

The objectives of this study were to further characterize the DHM-1 enrichment culture with respect to its ability to biodegrade CF at concentrations up to 4000 mg/L, CT (15 mg/L), and CFC-11 (25 mg/L), individually and in mixtures; to evaluate the effect of B_12_ dose and inoculum level on the maximum rate of CF biodegradation; to determine the effect of pH on the rate of CF biodegradation, and to evaluate the fate of CFC-11 using a fluoride mass balance.

## Materials and methods

### Inoculum, chemicals and MSM

Experiments were performed with the DHM-1 enrichment culture (ATCC no. PTA-120292) grown on corn syrup (regular type, Sweetener Products Company, Vernon, CA) and supplemented with cyanocobalamin (i.e., vitamin B_12_, USP grade; Research Organics, Inc., Cleveland, OH). The bicarbonate-buffered, sulfide-reduced MSM used to grow DHM-1 is described elsewhere (Shan et al. [2010a]).

Stock solutions of [^14^C]CT and [^14^C]CF were prepared in distilled deionized water at concentrations of approximately 4.5 μCi per mL, using neat [^14^C]CT (1.0 mCi/mmol) and [^14^C]CF (0.5 mCi/mmol) from American Radiolabeled Chemicals, Inc. (Saint Louis, MO). CT (99.9%, Sigma-Aldrich, Saint Louis, MO), CF (99.7%, Sigma-Aldrich), DCM (99.9%, AlliedSignal, Morristown, NJ), CFC-11 (99%, Sigma-Aldrich), and carbon disulfide (CS_2_, 100%, J.T. Baker, Center Valley, PA) were obtained as neat liquids. CM (99.9%, Praxair, Danbury, CT), HCFC-21 (dichlorofluoromethane, 99%, SynQuest Labs, Alachua, FL), HCFC-31 (chlorofluoromethane, 99%, SynQuest Labs), and methane (99.99%, Matheson, Longmont, CO) were obtained as neat gases. All other chemicals used were reagent grade.

#### Transformation of individual halomethanes

The ability of the DHM-1 enrichment culture to biodegrade individual halomethanes (~15 mg/L CT, 25 mg/L CFC-11, or 500–4000 mg/L CF) was evaluated with an initial inoculum of 2.5-5.0 mg/L protein (except in experiments that evaluated lower inoculum levels). Treatments were prepared with B_12_ (0.03 mol per mol of halomethane, except in experiments that evaluated lower inoculum levels), and without B_12_, in 160 mL serum glass bottles with 100 mL of MSM, in an anaerobic chamber (Coy Laboratory Products, Inc.) containing an atmosphere of approximately 98% N_2_ and 2% H_2_. The initial dose of corn syrup was 900 mg/L (~960 mg/L as chemical oxygen demand) (Shan et al. [2010a]). After purging the headspace with 30% CO_2_/70% N_2_ for 1 min, the bottles were sealed with 20-mm Teflon-faced red rubber septa and aluminum crimp caps. [^14^C]CT and [^14^C]CF were purified on a gas chromatograph prior to addition to the serum bottles to provide an initial ^14^C activity of 0.45 μCi/bottle, as previously described (Shan et al. [2010a]). The fate of CFC-11 was assessed based on release of fluoride instead of using [^14^C]CFC-11, which was prohibitively expensive.

Non-labeled CF, CT and CFC-11 were added using neat compounds. Media controls (no substrate, no culture) with and without B_12_ were prepared for each halomethane. The bottles were incubated quiescently in an inverted position at room temperature (22-24°C) in the anaerobic chamber. pH was monitored weekly and maintained between 6.7-7.7. Decreases in pH indicated that the corn syrup was undergoing fermentation; when decreases in pH stopped, a second dose of corn syrup was added.

The highest concentration of CF tested with DHM-1 in previous investigations was 500 mg/L (Shan et al. [2010a]). Experiments in this study evaluated CF concentrations of 1000, 2000, and 4000 mg/L. Serum bottles were prepared as described above, except that only unlabeled CF was added. Most experiments included water controls (WC), consisting of 100 mL of distilled deionized water and CF, CT, and CFC-11.

### Effect of B_12_ concentration and DHM-1 inoculum

The effect of vitamin B_12_ concentration on CF transformation rates by DHM-1 was evaluated in serum bottles as described above, except that the concentration of B_12_ was varied from 0.0 to 0.03 mol B_12_ per mol of CF added (500 mg CF/L = 4.19 mM), only one dose of corn syrup was added, and the bottles were continuously mixed on a shaker table. Media controls were included to evaluate abiotic losses of CF. The highest CF biodegradation rate for a given B_12_ dose was determined by linear regression of CF concentration versus time. The results for all B_12_ doses were fit (using Matlab, version 7.10.0) to a modified form of the Michaelis-Menten model:

(1) $$V=\frac{\mathrm{Vmax}\cdot \frac{B_{12}}{C_{0}}}{\frac{B_{12}}{K_{M}}+\frac{B_{12}}{C_{0}}}$$

where *V* = rate of CF biodegradation (mg/L/d); *V*_*max*_ = maximum rate of CF biodegradation (mg/L/d); *B*_*12*_*/C*_*o*_ = molar concentration of B_12_ added, divided by the molar concentration of CF added; and *B*_*12*_*/K*_*M*_ = molar ratio at which *V* is one half of *V*_*max*_.

The effect of DHM-1 inoculum concentration on the CF biodegradation rate was evaluated using 10 treatments. Half of the treatments received varying inoculum levels of DHM-1; the others consisted of abiotic controls. Treatments were prepared in the same manner described in the section for transformation of individual halomethanes, with an initial CF addition of approximately 500 mg/L (without [^14^C]CF).

### Effect of pH

Biodegradation rates for 500 mg/L of CF by DHM-1 were measured at pH levels from 5.0 to 7.7. B_12_ (0.03 mol B_12_ per mol of CF added) and corn syrup (900 mg/L) were added to all treatments. Serum bottles were prepared as described above for the B_12_ dose experiment, with the following modifications. The MSM was prepared at the target pH by varying the amounts of K_2_HPO_4_ and KH_2_PO_4_. After adding sodium sulfide, the final pH was adjusted using either H_3_PO_4_ (1 M) or NaOH (8 M); the MSM was incubated for six days to ensure equilibrium was reached at the target pH, before inoculating the DHM-1 enrichment culture (5% v/v). It was not necessary to sparge the headspace of the bottles with 30% CO_2_/70% N_2_. Each time CF was analyzed on the gas chromatograph, the pH was measured (0.2 mL sample) and, as needed, increased back to the target level using NaOH (8 M); decreases in pH were caused by fermentation of the corn syrup to organic acids. The highest CF biodegradation rate at a given pH was determined in the same manner described above for varying B_12_ doses. Lag times were based on the time from day zero to the first data point used to determine the highest biodegradation rate.

### Transformation of mixtures of halomethanes

The ability of DHM-1 to biodegrade mixtures of CT, CF, and CFC-11 was evaluated in mixtures of two or three compounds. With two compounds, a single dose of B_12_ was provided at the start (0.03 mol B_12_ per mol of total halomethanes added). When all of the halomethanes were present, B_12_ was added in a stepwise manner, i.e., the first dose of B_12_ was made based on the initial moles of CT; when CT transformation was nearly complete, a second dose of B_12_ was made based on the initial moles of CFC-11, and when CFC-11 was nearly consumed, a third dose was added based on the initial moles of CF. Along with the second dose of B_12_, the bottles were reinoculated with DHM-1 (i.e., another 5 μg protein per mL), based on preliminary tests that indicated the culture’s activity on CFC-11 and CF diminished after completing transformation of CT, presumably because CT transformation yields inhibitory intermediates (Lewis et al. [2001]). Controls with CT, CF and CFC-11 present included MSM + B_12_ added (but not inoculated), autoclaved (AC; inoculated with DHM-1 in MSM and then autoclaved for 1 h), and water only.

### Analytical methods and ^14^C distribution

The amounts of CT, CFC-11, CF, DCM, CM, HCFC-21, HCFC-31, methane and CS_2_ present in serum bottles were determined by analysis of headspace samples using a gas chromatographic method (Shan et al. [2010a,b]). Aqueous phase concentrations were calculated using Henry’s Law constants (Shan et al. [2010a,b]). Fluoride was measured by ion chromatography (details in Additional file 1). The amount of ^14^C activity and its distribution in the gas phase (quantified using gas chromatography followed by combustion) and liquid phase were determined as previously described (Shan et al. [2010a,b]). Protein concentration was measured with a BCA™ protein assay kit (Pierce Chemical Company) by following the manufacturer’s enhanced protocol after lysing the cells (Coleman et al. [2002]).

## Results

### Transformation of individual halomethanes

**Figure 1:**
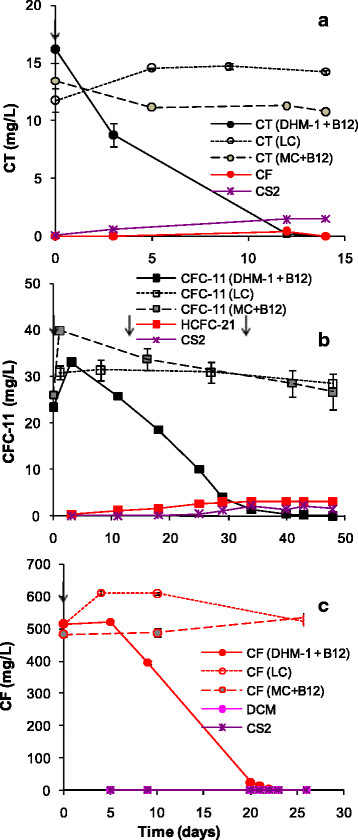
**Transformation of (a) CT; (b) CFC-11; and (c) CF, by DHM-1 in MSM with corn syrup and B**_**12**_**added.** LC = live control (DHM-1 with corn syrup but without B_12_); MC + B_12_ = media control with B_12_. Daughter products are shown only for the DHM-1 + B_12_ treatments. ↓ = addition of corn syrup; B_12_ was added only at t = 0. Error bars are the data range for duplicate bottles; when not visible, the bars are smaller than the symbols. The initial increase in CFC-11 was likely due to insufficient time to establish equilibrium at time zero between the headspace and liquid phases.

When provided with corn syrup and B_12_, DHM-1 readily biodegraded CT, CFC-11, and CF (Figure 1). Average transformation rates [i.e., (initial concentration)/(time to reach the detection limit)] were 1.3, 0.54, and 22 mg/L/d for CT, CFC-11, and CF, respectively. Reductive dehalogenation products (i.e., CF, DCM and CM from CT; HCFC-21 from CFC-11; and DCM and CM from CF) at the end of the incubation period represented 6% or less of CFC-11 and 1% percent of CT and CF. CS_2_ accounted for 5-16% of the CT consumed, indicating a substitutive pathway was involved. Over a 14 day period, approximately 20% of the initial CT was consumed in the media + B_12_ treatment. No losses occurred in the live control (i.e., DHM-1 with corn syrup but without B_12_) or in media without B_12_ (Additional file 1: Figure S1), indicating that abiotic transformation of CT in MSM was mediated by B_12_. Biotransformation of CFC-11 started after a lag of approximately 10 days and was complete by day 48 (Figure 1b). Approximately equal amounts of HCFC-21 and CS_2_ (i.e., 3 μmol/bottle each) accumulated, while formation of HCFC-31 was negligible. Only a minor amount of CFC-11 transformation occurred in the live control and media + B_12_ control. No transformation of CFC-11 occurred in the MSM control without B_12_ (Additional file 1: Figure S1). Biotransformation of CF (513 mg/L) occurred only in the live treatment with DHM-1, corn syrup, and B_12_; none was observed in the live control, media + B_12_ control, or in the media without B_12_ control (Additional file 1: Figure S1). Methane formation was absent in all treatments.

^14^CO and ^14^CO_2_ were the dominant products from transformation of [^14^C]CT and [^14^C]CF (Table 1). The sum of CO and CO_2_ accounted for approximately 70% of CT or CF transformation. CO_2_ predominated from CT transformation (i.e., over 50%) while CO was predominant from CF transformation. Only a minor amount of CS_2_ accumulated from CT transformation (i.e., 5.2%). Soluble compounds were the third most significant ^14^C-labeled product. High performance liquid chromatographic analysis indicated that the main products were formate (35-36%) and propionate (14-23%) (Additional file 1: Table S1). Synthesis of propionate from CO_2_ has been observed previously, via pathways speculated to include a reversal of syntrophic propionate degradation or reductive formation from H_2_ + CO_2_ in combination with homoacetogenesis (Conrad and Klose [1999]).

**Table 1:** **Products from biodegradation of [**^**14**^**C] CT and [**^**14**^**C] CF by DHM-1**

^**14**^**C added as:**	**% of**^**14**^**C added recovered as**^***a***^										
	**CH**_**4**_	**CO**	**CM**	**DCM**	**CS**_**2**_	**CF**	**CT**	**other**^***b***^	**CO**_**2**_	**Soluble**^***c***^	**Loss**^***d***^
CT	0.1	**14**	0.3	0	5.2	2.2	0	0.1	**51**	**27**	0
CF	0.5	**67**	0	0	0	0.9	0.2	0.8	2.7	**15**	**13**

^*a*^Numbers in bold represent the main products formed.

^*b*^Other volatile ^14^C-labeled compounds that were not identified.

^*c*14^C-labeled compounds that remained after sparging samples under acidic conditions; HPLC analysis indicated the majority of this fraction consisted of organic acids (see text).

^*d*^Loss = amount of ^14^C added that was not accounted for in the other compounds or categories listed.

A mass balance for fluoride release during biodegradation of CFC-11 by the DHM-1 enrichment culture was evaluated. In the presence of corn syrup and B_12_, the average fluoride recovery was 99.5% (Table 2). This takes into account the fluoride that resided in the minor amount of HCFC-21 and HCFC-31. Thus, nearly all of the CFC-11 consumed by DHM-1 in the presence of corn syrup and B_12_ resulted in stoichiometric release of fluoride. The increase in fluoride coincided with consumption of CFC-11 (Additional file 1: Figure S2). Fluoride was also detected in the abiotic control containing MSM and B_12_, although only 44% as much CFC-11 was degraded and the percent recovery for fluoride was not as high. There was no significant consumption of CFC-11 or release of fluoride in the treatments without B_12_ added.

**Table 2:** **Fluoride mass balance from degradation of CFC-11**^***a***^

**Treatment**	**No. of bottles**	**CFC-11 consumed (μmol/bottle)**	**F**^**−**^**released (μmol/bottle)**	**CHCl**_**2**_**F formed (μmol/bottle)**	**CH**_**2**_**ClF formed (μmol/bottle)**	**F**^**−**^**recovery (%)**^***b***^
DHM-1 + CS + B_12_^*c*^	14	61.6 ± 2.1^*d*^	57.4 ± 4.6	3.1 ± 1.0	0.8 ± 0.9	99.5 ± 8.0
DHM-1 + CS^*e*^	3	1.8 ± 1.2	0	0.9 ± 0.1	0.09 ± 0.04	-
MSM + B_12_	6	27.1 ± 2.7	17.7 ± 5.0	0.5 ± 0.3	0.06 ± 0.05	68.0 ± 10.1
MSM	6	1.5 ± 3.0	0	0.06 ± 0.03	0.2 ± 0.3	-

^*a*^Headspace monitoring results are presented in the Supporting Information.

^*b*^Calculated according to equation S-1 in Additional file 1.

^*c*^Includes three bottles that also received 500 mg/L CF, which had no effect of the F^-^ mass balance, CS = corn syrup.

^*d*^ ± = Standard deviation.

^*e*^Inoculum was washed in fresh MSM to avoid carryover of B_12_.

**Figure 2:**
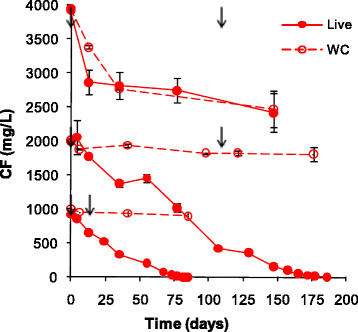
**CF biodegradation by DHM-1 in MSM with one dose of B**_**12**_**(at t = 0) and two doses of corn syrup (arrows); WC = water control.** Error bars represent the data range for duplicate bottles; when not visible, the bars are smaller than the symbols.

The ability of the DHM-1 enrichment culture to biodegrade CF concentrations above 500 mg/L was evaluated. Approximately 1000 mg/L (i.e., 900 μmol/bottle) was transformed in 85 days and 2000 mg/L (i.e., 1800 μmol/bottle) in 180 days (Figure 2). Accumulation of DCM and CS_2_ was negligible (<0.5 and 1.0 μmol/bottle, respectively). WC results indicated that diffusive loss of CF was minor. Activity on CF ceased at 4000 mg/L (i.e., 3600 μmol/bottle), which is approximately 50% of the aqueous solubility of CF at 20°C. Growth of DHM-1 on corn syrup, however, was not adversely affected by the high concentrations of CF. Protein concentrations increased to 89, 97 and 128 μg/mL for treatments that received 1000, 2000 and 4000 mg/L of CF, respectively; this is similar to previously reported levels for DHM-1 in the absence of CF, and in the presence of CF at 500 mg/L (Shan et al. [2010a]). Although growth of DHM-1 in the presence of CT or CFC-11 was not monitored, an increase in the turbidity of the MSM a few days after inoculation suggested that DHM-1 also grows on corn syrup in the presence of CT and CFC-11.

### Effect of B_12_ concentration and pH on CF biodegradation by DHM-1

**Figure 3:**
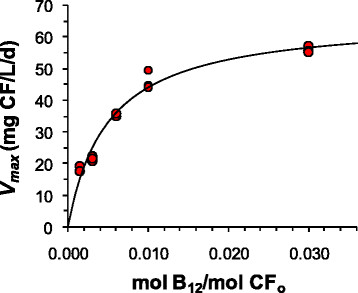
**Effect of vitamin B**_**12**_**concentration on maximum CF transformation rates by DHM-1; B**_**12**_**concentrations are expressed in terms of mol B**_**12**_**added per mol CF initially added (CF**_**o**_**).** The line represents the fit to equation 1.

Reducing the molar ratio of B_12_ added per mole of CF added from 0.03 mol B_12_ per mol of CF added to 0.01 resulted in a moderate decrease in the maximum CF transformation rate, while the rate fell more quickly below 0.01 (Figure 3). Fitting the data to equation 1 resulted in a *V*_*max*_ of 66 ± 4.6 mg CF/L/d and a *B*_*12*_/*K*_*m*_ ratio of 0.0050 ± 0.0010 mol B_12_ per mol CF (±values indicate 95% confidence intervals). Assuming a yield of 50–60 mg protein/L from the single dose of corn syrup added (Shan et al. [2010a]), *V*_*max*_ can be normalized to approximately 1.2 mg CF/mg protein/d.

**Figure 4:**
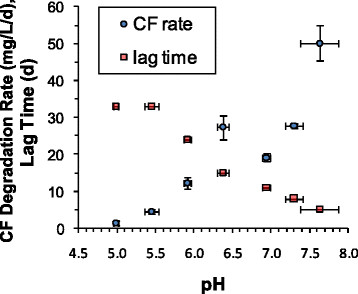
**Impact of pH on biodegradation rates for CF by DHM-1 and the length of the lag phase prior to the onset of biodegradation.** Error bars in both directions indicate the 95% confidence interval, based on results from triplicate bottles at each pH.

Maximum CF biodegradation rates for DHM-1 increased with increasing pH from 5.0 to 7.7 (Figure 4). There appeared to be a plateau in the pH range from 6.4 to 7.3, while the rate at pH 7.7 almost doubled relative to that in the circumneutral pH region, reaching 50 mg/L/d. The activity of DHM-1 diminished substantially below pH 6.0 and ceased at pH of 5.0. Lag times (i.e., the time prior to the onset of a maximum rate) decreased as pH increased.

### Effect of DHM-1 inoculum level on CF biodegradation

**Figure 5:**
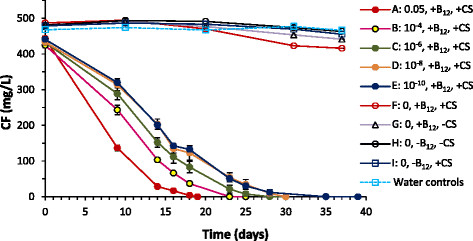
**CF degradation with different inoculum levels of DHM-1 (percent by volume, indicated by the number after the treatment letter).** Treatments A through E contained DHM-1, corn syrup (CS) and B_12_ (3 mol% of CF). Treatments F through I were abiotic controls. Error bars show standard deviations of triplicate bottles.

Biodegradation of CF proceeded at a high rate even at a DHM-1 inoculum level as low as 10^−8^ percent (v/v) (Figure 5, treatments A-E). The 5% inoculum (v/v) corresponds to a protein concentration of approximately 5 mg/L, so the 10^−8^ inoculum equates to approximately 10^-8^ mg/L. Headspace monitoring continued until CF fell below the maximum contaminant level (MCL) for trihalomethanes (80 μg/L). At a 5% inoculum inoculum level, 23 days of incubation was required, while 39 days was required for the 10^−8^% inoculum level. Correspondingly, the maximum initial degradation rate was approximately twice as high at the highest inoculum (30 mg/L/d) compared to the lowest (17 mg/L/d). Accumulation of DCM and CM amounted to less than 0.6% of the CF consumed. Losses from uninoculated controls (treatments F-I) were comparatively minor. These results demonstrate the potential for DHM-1 to biodegrade high concentrations of CF even at a low initial cell density, which is an essential characteristic for use in bioaugmentation.

### Biodegradation of mixtures of halomethanes

**Figure 6:**
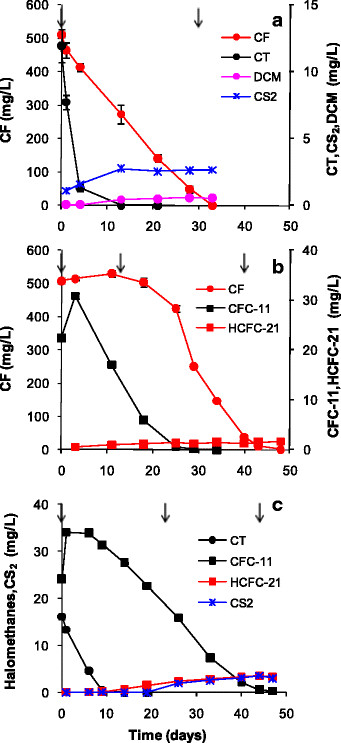
**Performance of DHM-1 in transforming mixtures of (a) CT + CF; (b) CFC-11 + CF; and (c) CT + CFC-11 in MSM, with corn syrup and B**_**12**_**added (t = 0).** Arrows indicate addition of corn syrup. Error bars are the data range for duplicate bottles; when not visible, the bars are smaller than the symbols.

In the presence of 12 mg/L CT (13 μmol/bottle) and 512 mg/L CF (463 μmol/bottle), DHM-1 biodegraded both halomethanes simultaneously, with no apparent effect of CT on CF or vice versa (Figure 6a). A low amount of CS_2_ (5.2 μmol/bottle) accumulated, accounting for 1% of the CT + CF transformed. Formation of DCM (0.6 μmol/bottle) was negligible. With a mixture of 23 mg/L CFC-11 (55 μmol/bottle) and 509 mg/L CF (461 μmol/bottle), DHM-1 biodegraded CFC-11 faster than when CFC-11 was added individually (Figure 1b versus 6b), possibly related to changes in membrane fluidity or homeoviscous and homeophasic adaptation during growth in the presence of a high concentration of CF (Shan et al. [2010a]). In contrast, CF transformation was inhibited by the presence of CFC-11; CF transformation did not begin until the concentration of CFC-11 dropped to 6 mg/L (14 μmol/bottle) on day 18. Minor amounts of CS_2_, DCM and HCFC-21 accumulated in comparison to the amount of halomethanes removed. With a mixture of 16 mg/L CT (17 μmol/bottle) and 24 mg/L CFC-11 (57 μmol/bottle), CT was consumed in 10 days; following a lag phase of approximately 10 days, CFC-11 was consumed by day 47 (Figure 6c). These patterns are similar to what occurred with the individual compounds, indicating no apparent interaction between CT and CFC-11. The combination of CT and CFC-11 resulted in more CS_2_ accumulation than the other two-component mixtures, accounting for 7.7% of the CT and CFC-11 transformed. Formation of HCFC-21 was also slightly higher than in the mixture of CFC-11 and CF, while formation of DCM was negligible.

**Figure 7:**
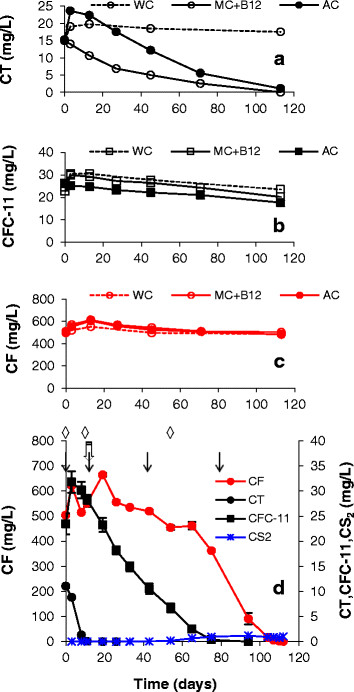
**Behavior of a mixture of halomethanes in controls (a, b and c) and (d) live bottles with DHM-1 and corn syrup + B**_**12**_**in MSM.** ↓ = addition of corn syrup; ◊ = addition of B12; ⇩ = reinoculation with DHM-1; WC = water controls, MC + B12=mediacontrol with B12, and AC = autoclaved control with B12. Averages forduplicate bottles are shown. Error bars in panel d represent the datarange; when not visible, the bars are smaller than the symbols.

When CT, CFC-11, and CF (11, 24, and 500 mg/L, respectively) were added at the same time, the pattern of transformation was similar to the two-component mixtures. CT (12 μmol/bottle) transformation was completed first, followed by CFC-11 (57 μmol/bottle) and then CF (456 μmol/bottle) (Figure 7d). One difference between the live treatments shown in Figures 6 and 7d is the pattern of B_12_ addition. With the two-component mixtures (Figure 6), B_12_ was added only at the start. With the three-component mixture (Figure 7d), the same molar ratio of B_12_ was applied, although the additions were timed to coincide with the beginning of transformation of each compound. Accumulation of CS_2_, DCM, CM, HCFC-21, and HCFC-31 was negligible. Results for controls are shown in Figure 7, panels a, b and c. Nearly complete transformation of CT occurred by day 113 in the media control and AC, versus no significant losses from the WC. Approximately 33% and 11% of CFC-11 was removed in the autoclaved and media controls, respectively, while loss of CFC-11 in WC was minor. Losses of CF in all of the controls were minor. These results demonstrated that transformation of CF was exclusively a biotic process, while abiotic processes contributed to transformation of CT and CFC-11. However, transformation of CT and CFC-11 was considerably faster in the presence of live cells, and DHM-1 was able to achieve complete transformation of a mixture of CT, CFC-11 and CF at high initial concentrations in less than four months in MSM.

## Discussion

The feasibility of using bioremediation to clean up halogenated solvents in the vicinity of nonaqueous phase liquids depends in part on the ability of microbes to grow in the presence of contaminant concentrations that approach their aqueous solubility limit. The results of this study indicate that the DHM-1 enrichment culture grows in the presence of at least 4000 mg/L CF, approximately one half its aqueous solubility, and the culture retains its ability to biodegrade CF at 2000 mg/L. When transforming 500 mg/L of CF, the two *Pantoea* spp. isolated from DHM-1 adapted their growth by alteration of their membrane fluidity or homeoviscous and homeophasic adaptation (Shan et al. [2010a]). A similar response at the higher CF concentrations evaluated in this study seems likely. Tolerance of high concentrations of halogenated compounds appears to be a characteristic of *Enterobacter* species (Sharma and McCarty [1996]), which are closely related to *Pantoea* (Shan et al. [2010a]). Several bioaugmentation cultures that are enriched in *Dehalococcoides* and used to treat chlorinated ethenes also possess the ability to grow at the high solvent concentrations found near nonaqueous phase liquids (ESTCP 2005).

DHM-1 was effective in transforming CT (~11 mg/L) and CFC-11 (~24 mg/L) as well as CF. Based on experiments with ^14^C-labeled CT and CF, the primary transformation products are environmentally benign (CO_2_, CO and organic acids). Similar results were obtained with DHM-1 when it was in an earlier stage of development (Shan et al. [2010b]). Although [^14^C]CFC-11 was not used in this study, the majority of the CFC-11 consumed was accounted for as fluoride and no volatile products were detected, suggesting the primary transformation products were nonhazardous. DHM-1 did not produce significant levels of DCM and CM from CT and CF. DCM and CM are not necessarily problematic, since both are readily fermentable (Mägli et al. [1998]). In contrast, little is known about the anaerobic biodegradability of HCFC-21 and HCFC-31; if significant levels are formed from CFC-11, it is unclear how quickly they can be degraded. Preliminary experiments indicate an anaerobic culture that grows on DCM as its sole substrate was unable to utilize HCFC-31 (Shan [2009]).

Halomethanes are often found in mixtures at hazardous waste sites. DHM-1 was effective in transforming mixtures of CT, CFC-11 and CF. Consistent with general expectations for halomethanes (Wackett et al. [1992]), CT was transformed first, followed by CFC-11 and then CF. The inhibitory effect of CFC-11 on CF is a potential concern, since the average rate of CFC-11 transformation by DHM-1 was approximately 40-fold slower than CF.

An important consideration for cultures used in bioaugmentation is the required inoculum level. For chlorinated ethenes, a commonly used target is 10^7^ cells/L of *Dehalococcoides* (ESTCP [2010]). Assuming a unit mass of 1.6 × 10^−14^ g/cell (Cupples et al. [2003]) and one half of the cell mass is protein, this equates to an inoculum of 8 × 10^−5^ mg/L protein. DHM-1 was able to biodegrade 500 mg/L of CF at a high rate with a volumetric addition as low as 10^−8^ percent; this equates to a concentration of 10^−8^ mg/L protein, several orders of magnitude lower than the target for *Dehalococcoides*. This suggests that achieving an adequate inoculum in situ will not be problematic.

The catalytic degradation of halogenated compounds by B_12_ and other transition metal coenzymes under low redox conditions has long been recognized (e.g., Gantzer and Wackett [1991]; Wackett et al. [1992]). One of the concerns with using B_12_ along with DHM-1 for bioaugmentation is the culture’s relatively high requirement for B_12_. In a previous study we used a B_12_ molar dose of 3%, i.e., 0.03 mol B_12_ per mol CF (Shan et al. [2010a]). Varying the B_12_ dose in this study indicated a half saturation value of 0.005 mol B_12_/mol CF. A ratio 0.005 mol B_12_/mol CT also significantly improved the rate of CT degradation in a methanogenic sludge consortium, at a CT concentration of 15.4 mg/L (Guerrero-Barajas and Field [2005]). Nevertheless, for halomethane concentrations in the hundreds of mg/L, the B_12_ required by DHM-1 is several orders of magnitude higher than what is needed to grow *Dehalococcoides* (He et al. [2007]), for which B_12_ functions as a cofactor for reductive dehalogenases (Schipp et al. [2013]).

Aquifer pH is a significant concern for bioaugmentation, since many cultures lose effectiveness at pH levels below 6 or above 8. The highest pH evaluated in this study was 7.6, which yielded a higher rate than in the circumneutral pH region (6.3-7.3; Figure 4). CF biodegradation rates decreased significantly below 6.0 and activity essentially ceased at pH 5.0. This is similar to the behavior of many *Dehalococcoides* enrichment cultures (ESTCP 2005; Vainberg et al. [2009]). The difficulties associated with adjusting aquifer pH include non-homogenous distribution of the buffering agent and the potential for clogging due to precipitation when pH is increased.

For contaminant plumes with high concentrations of halomethanes that are not undergoing natural attenuation, the options for bioremediation are limited. The benefits of using a culture such as DHM-1 include its high rate of CF transformation; its ability to transform mixtures of CT, CF, and CFC-11; the conversion of these halomethanes to environmentally benign products; its growth on an inexpensive primary substrate (corn syrup); and its ability to grow in the presence of CF at levels at least as high as 4000 mg/L. The culture also retains its ability to anaerobically transform CF after exposure to air for as long as one day (Additional file 1: Figure S3). Further studies are needed to validate the use of DHM-1 under field conditions, and to determine if lower cost formulations of B_12_ can be developed, e.g., using the fermentation product from cultures that synthesize B_12_ with a lesser degree of purification.

## Competing interests

The authors declare that they have no competing interests.

## Authors’ contributions

HS carried out the experiments to evaluate biodegradation of CT, CF, and CFC-11, individually and in mixtures, as well as the biodegradability of CF at concentrations above 500 mg/L. HW and RY performed experiments on the effect of pH and B_12_ on the biodegradation rate for CF, including the parameter estimation for equation 1. PJ carried out experiments to determine the mass balance on fluoride during biodegradation of CFC-11 and the effect of inoculum level on the rate of CF biodegradation. DLF conceived of the study, and participated in its design and coordination and helped to draft the manuscript. All authors read and approved the final manuscript.

## Additional file

## Supplementary Material

Additional file 1: **Fluoride Measurements; Abiotic Controls Figure S1.** Soluble Products from Biodegradation of CT and CF **Table S1.** Fluoride Mass Balance **Figure S2.** and Ability of DHM-1 to Tolerate Exposure to Oxygen **Figure S3.** Click here for file
