# Supplementary material for: Biodegradation of high concentrations of halomethanes by a fermentative enrichment culture
Source: AMB Express. 2014 Jun 14;4:48. doi: 10.1186/s13568-014-0048-5 (PMC4230812; doi:10.1186/s13568-014-0048-5)
Supplement: Additional file 1: — Fluoride Measurements; Abiotic Controls Figure S1. Soluble Products from Biodegradation of CT and CF Table S1. Fluoride Mass Balance Figure S2. and Ability of DHM-1 to Tolerate Exposure to Oxygen Figure S3. [file s13568-014-0048-5-S1.docx]

**Additional file 1**

**Supplementary Material for Fluoride Measurements**

Fluoride concentrations were quantified using a Dionex 2100 Ion Chromatograph (IC). A mobile phase of degassed sodium carbonate (4.5 mM)/bicarbonate (0.8 mM) was pumped (1.0 mL/min) through an IonPac® AG9-HC guard column (4 mm x 50 mm), followed by an IonPac® AS9-HC anion-exchange column (4 mm x 250 mm. Samples (0.2 mL). Samples were prepared by a 1:50 dilution in DDI water, followed by filtration (0.2 µm PTFE). Calibration standards were prepared according to EPA method 9214. The percent recovery of fluoride (*R_F_*) was calculated as follows:

|  | $R_{F}=\frac{(F_{f}-F_{i})\cdot V_{l}}{{CFC11}_{i}-{CFC11}_{f}-{HCFC21}_{f}-{HCFC31}_{f}} \times100$ | (S-1) |
| --- | --- | --- |

where *F_f_* and *F_i_* = the final and initial fluoride concentrations (µM), respectively; *V_l_* = volume of liquid (0.10 L/bottle); *CFC*11*_i_* and *CFC*11*_f_* = the initial and final amounts of CFC-11 (µmol/bottle), respectively; *HCFC*21*_f_* = final amount of dichlorofluoromethane (µmol/bottle); and *HCFC*31*_f_* = final amount of chlorofluoromethane (µmol/bottle). The initial amounts of HCFC-21 and HCFC-31were zero.

**Supplementary Material for Abiotic Controls**

In the first paragraph of the Results, mention is made of abiotic controls for CT, CFC-11 and CF that contain mineral salts medium (MSM) without vitamin B_12_. Figure S-1 presents these results. As stated in the text, there was no significant decrease in any of the compounds under these abiotic incubation conditions in the absence of B_12_.

Figure S1 Mineral salts media controls for CT, CFC-11 and CF in the absence of B_12_

**Supplementary Material for Soluble Products from Biodegradation of CT and CF**

In the second paragraph of the Results, mention is made of soluble products from biodegradation of [^14^C]CT and [^14^C]CF. At the end of the incubation period, serum bottles were analyzed for the distribution of ^14^C-labeled products. Part of this analysis included sparging samples from the bottles with N_2_ under acidic conditions. The liquid remaining (which we refer to as nonstrippable residue, NSR) was analyzed for total ^14^C activity and then was subjected to fractionation by high performance liquid chromatography (HPLC). Fractions that eluted from the HPLC eluent were collected, corresponding to the retention times for several organic acids and ethanol, and then analyzed for ^14^C activity. The complete results are shown in Table S1. As stated in the text, the main ^14^C-labeled products in [^14^C]NSR were formate (34.6-36.0%) and propionate (13.5-22.9%).

**Table S1 Organic acid analysis of ^14^C-labeled NSR from biodegradation of CF and CT by DHM-1**

|  | % of [^14^C]NSR from [^14^C]CF | | | % of [^14^C]NSR from [^14^C]CT | | |
| --- | --- | --- | --- | --- | --- | --- |
| Compound *^b^* | #1 | #2 | Ave | #1 | #2 | Ave |
| UN #1*^a^* | 0.0 | 2.0 | 1.0 | 3.9 | 3.0 | 3.5 |
| UN #2 | 3.0 | 1.4 | 2.2 | 0.0 | 1.7 | 0.9 |
| lactate | 2.4 | 0.5 | 1.4 | 0.7 | 0.0 | 0.3 |
| formate | 39.2 | 32.9 | 36.0 | 34.0 | 35.2 | 34.6 |
| acetate | 3.0 | 1.8 | 2.4 | 9.7 | 2.5 | 6.1 |
| UN #3 | 4.9 | 1.2 | 3.1 | 1.8 | 0.7 | 1.2 |
| propionate | 12.6 | 14.4 | 13.5 | 23.7 | 22.2 | 22.9 |
| ethanol | 5.7 | 4.6 | 5.1 | 2.0 | 1.4 | 1.7 |
| isobutyrate | 2.8 | 3.5 | 3.2 | 1.8 | 2.1 | 1.9 |
| butyrate | 0.0 | 0.0 | 0.0 | 1.9 | 1.0 | 1.4 |
| UN #4 | 7.5 | 4.7 | 6.1 | 3.0 | 1.2 | 2.1 |

*^a^* Compounds are listed in the order in which they eluted off the HPX-87H HPLC column.

*^b^* UN = unknown; the identity of the compound that eluted in this fraction was not determined.

**Supplementary Material for the Fluoride Mass Balance**

In the third paragraph of the Results, mention is made of fluoride release during CFC-11 biodegradation. Three sets of experiments were performed to evaluate a mass balance for fluoride. For two of the sets, fluoride was measured only at the start and end of the incubation period. For one set, fluoride was measured at multiple time points as CFC-11 decreased. Headspace monitoring and fluoride results for this set are shown in Figure S2, for the treatment that received DHM-1, CS and B_12_ (first row in Table 2), and for the treatment with only MSM (last row in Table 2). It is apparent that fluoride release coincided with CFC-11 biodegradation in the treatment with DHM-1, CS and B_12_. Over the same period, the net decrease in CFC-11 (initial versus final value) was 6% in the treatment with only MSM present.


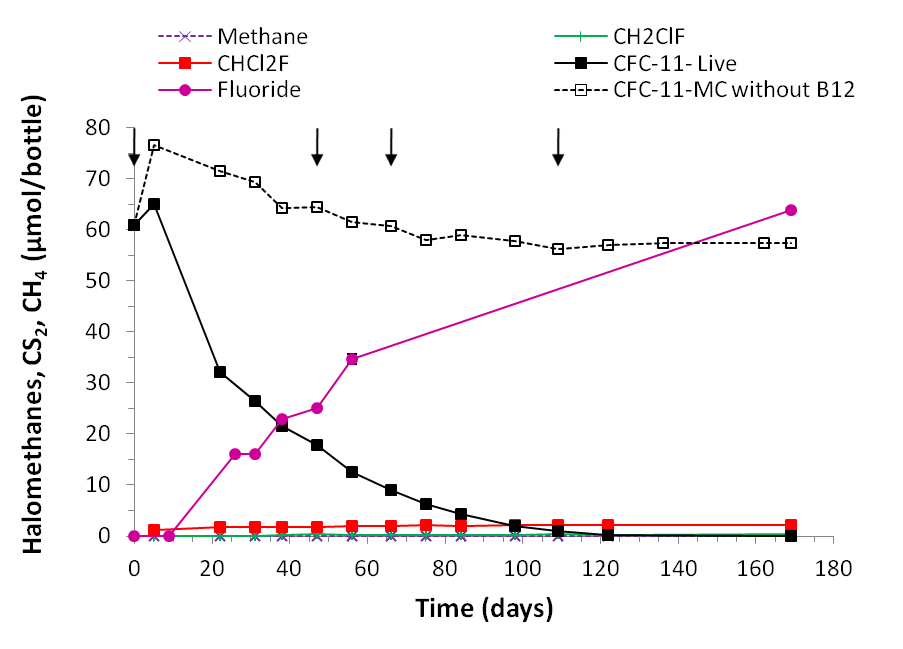


**Halomethanes, CH_4_, and F-**

**(µmol/bottle)**

**Figure S2** Live bottles contained DHM-1, corn syrup, B_12_ and CFC-11. The control (CFC-11-MC without B_12_) contained only MSM and CFC-11. CHCl_2_F, CH_2_ClF, CH_4_, and fluoride are for the live bottles only. Error bars represent standard deviations of triplicate bottles. Arrow indicate the addition of corn syrup to the live bottles

**Supplementary Material for the Ability of DHM-1 to Tolerate Exposure to Oxygen**

In the last paragraph of the Discussion, mention is made of DHM-1’s ability to anaerobically transform CF after exposure to air for as long as one day. Figure S3 presents this result.

**Figure S3** CF transformation by DHM-1 after 24 h exposure to air before returning to anaerobic MSM and adding corn syrup, in comparison bottles without prior exposure to air. Results represent the average of duplicate bottles. Arrows indicate addition of corn syrup
